# Three Methods of Estimating Mesophyll Conductance Agree Regarding its CO_2_ Sensitivity in the Rubisco-Limited C_i_ Range

**DOI:** 10.3390/plants7030062

**Published:** 2018-08-05

**Authors:** James Bunce

**Affiliations:** Adaptive Cropping Systems Lab and PP Systems, USDA-ARS, Haverhill, MA 01913, USA; jb@ppsystems.com; Tel.: +1-410-451-7343

**Keywords:** photosynthesis, stomatal conductance, internal CO_2_ concentration, chlorophyll fluorescence, mesophyll conductance

## Abstract

Whether the mesophyll conductance to CO_2_ movement (g_m_) within leaves of C_3_ plants changes with CO_2_ concentration remains a matter of debate, particularly at low CO_2_ concentrations. We tested for changes in g_m_ over the range of sub-stomatal CO_2_ concentrations (C_i_) for which Rubisco activity limited photosynthesis (A) in three plant species grown under the same conditions. Mesophyll conductance was estimated by three independent methods: the oxygen sensitivity of photosynthesis, variable J fluorescence combined with gas exchange, and the curvature of the Rubisco-limited A vs. C_i_ curve. The latter assay used a new method of rapidly obtaining data points at approximately every 3 μmol mol^−1^ for Rubisco-limited A vs. C_i_ curves, allowing separate estimates of curvature over limited C_i_ ranges. In two species, soybean and sunflower, no change in g_m_ with C_i_ was detected using any of the three methods of estimating g_m_. In common bean measured under the same conditions as the other species, all three methods indicated large decreases in g_m_ with increasing C_i_. Therefore, change in g_m_ with C_i_ in the Rubsico-limited region of A vs. C_i_ curves depended on the species, but not on the method of estimating g_m_.

## 1. Introduction

The importance of mesophyll conductance to CO_2_ movement (g_m_) within leaves of C_3_ species in limiting rates of photosynthesis (A) has become increasingly apparent [1,2,3]. Several basically different methods of estimating g_m_ have been developed, including on-line carbon isotope discrimination [4,5], two different types of chlorophyll fluorescence measurements combined with CO_2_ fixation rates [6], methods based on the curvature of initial A vs. C_i_ curves [7], and calculation based on the oxygen sensitivity of photosynthesis [8]. All of these methods are based on discrepancies between sub-stomatal CO_2_ (C_i_) values estimated by gas exchange and estimates of CO_2_ at the site of Rubisco inside the chloroplast (C_c_) based on the biochemical C_3_ photosynthesis model of Farquhar, von Caemmerer and Berry [9]. Several variations on some of the fluorescence-based methods have also been developed. Singh and Reddy [10] compared several methods and some of their variations in soybean leaves, and found little disagreement among the methods compared. Killi and Haworth [11] reported similar g_m_ values obtained from curvature of A vs. C_i_ curves and from fluorescence measurements combined with gas exchange. However, those comparisons did not deal with possible changes in g_m_ with CO_2_ concentration. Variation in g_m_ with measurement CO_2_ concentration has been reported in several cases [5,12,13,14,15,16], but was not found in others [4,6,16]. However, many of these measurements were limited to C_i_ values > 200 μmol mol^−1^ because lower C_i_ values increase errors in the estimate [6]. However, because photosynthesis would be most sensitive to variation in g_m_ in the Rubisco-limited region of low C_i_, this work focused on comparing methods in this region of response curves. Some modelling efforts suggested that some apparent variation in g_m_ with CO_2_ concentration could be artefacts caused by photorespiration [17]. However, Flexas et al. [12] and Mizokami et al. [18] reported changes in g_m_ with CO_2_ even at low O_2_, which eliminates photorespiration in these particular cases. Furthermore, an effect of CO_2_ concentration on aquaporins has been reported [19], potentially providing a mechanism for CO_2_ impacts on g_m_ [18].

In this paper, three different methods of estimating g_m_ were used to test for changes in g_m_ with C_i_ over the Rubisco-limited range of C_i_ in three different plant species. One of the methods used a new method of rapidly generating many densely positioned data points on A vs. C_i_ curves of single leaves.

## 2. Results

**Figure 1:**
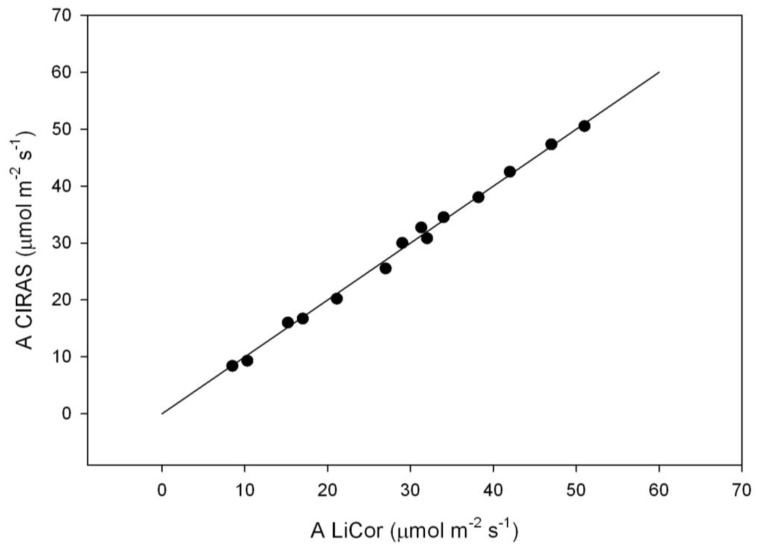
CO_2_ assimilation rates (A) of sunflower (*Helianthus annuus*) leaves at 2% O_2_ in N_2_ measured with a LiCor 6400 portable photosynthesis system, using the system corrections for O_2_ concentration, and measured with a CIRAS-3 portable photosynthesis system at the same sub-stomatal CO_2_ concentrations, without correction for O_2_ concentration. The line is the 1:1 line.

At 2% O_2_, values of A at each C_i_ corrected for the O_2_ sensitivity of water vapor and carbon dioxide analysis by the LiCor 6400 system software were in close agreement with values of A at the same C_i_ measured with the CIRAS-3 system without correction for O_2_ concentration (Figure 1).

**Figure 2:**
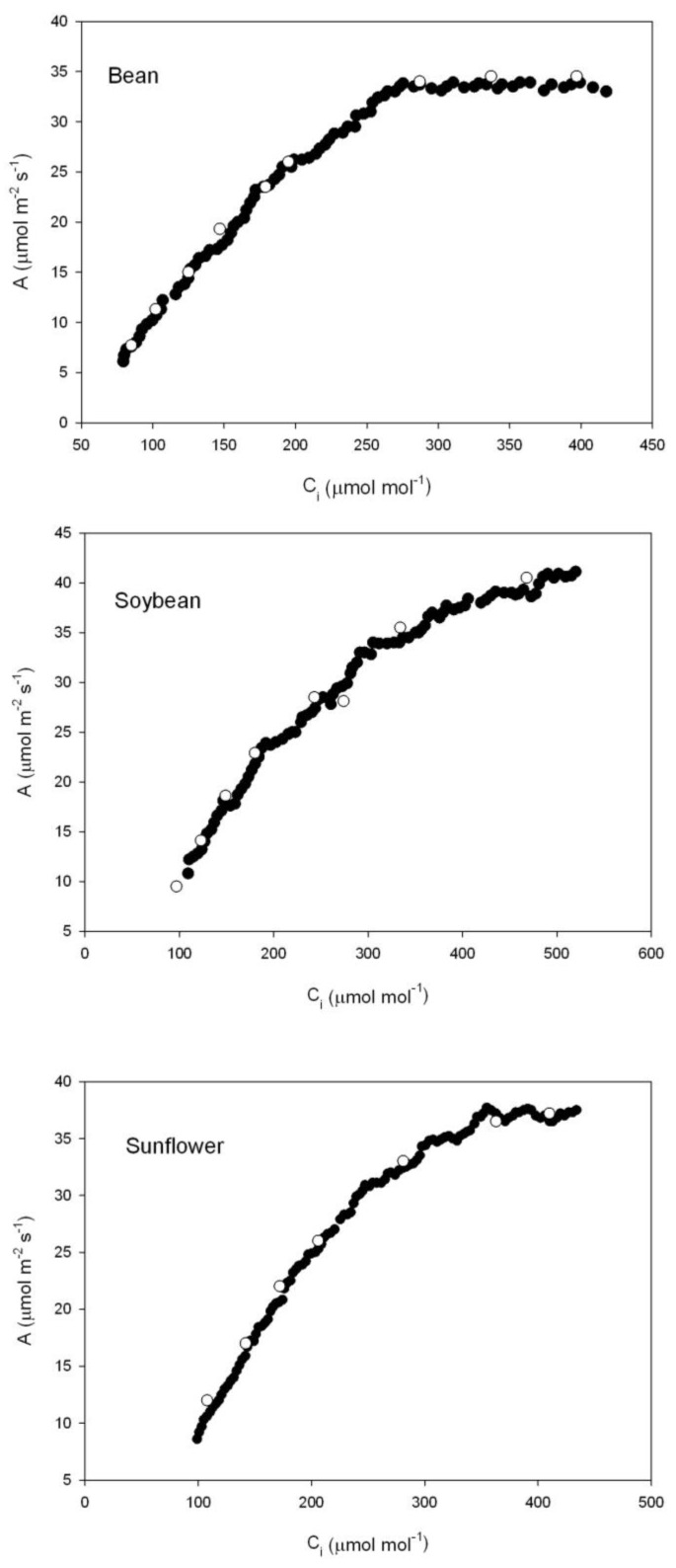
CO_2_ assimilation rate (A) over a range of sub-stomatal CO_2_ concentrations (C_i_) in single bean (*Phaseolus vulgaris*), soybean (*Glycine max*), and sunflower (*Helianthus annuus*) leaves. Open symbols: steady-state data points. Closed symbols: data points obtained from ramped CO_2_ on the same leaves. See text for details of methods.

Examples of multipoint A vs. C_i_ curves obtained with the CIRAS-3 CO_2_ ramping technique are presented in Figure 2. The data define reasonably smooth A vs. C_i_ curves, and agree well with steady-state A vs. C_i_ curves. There were enough data points generated rapidly from single leaves to use the Sharkey et al. [20] curve fitting program for at least three separate sections of the Rubisco-limited part of the curve. The analysis program generally requires at least five data points in the Rubisco-limited part of the curve.

In testing the sensitivity of the variable J fluorescence method of estimating g_m_ [6] to values of photorespiration at high C_i_, photorespiration was either estimated to be zero at the external CO_2_ concentration of 1800 μmol mol^−1^ or to be 2.5% of photosynthesis (see Section 4). The values of g_m_ in the C_i_ range of 100 to 200 μmol mol^−1^ estimated from combined gas exchange and fluorescence varied by only a few percent depending on the assumed values of photorespiration. At higher C_i_ values, those differences in g_m_ estimates would have been more substantial.

**Figure 3:**
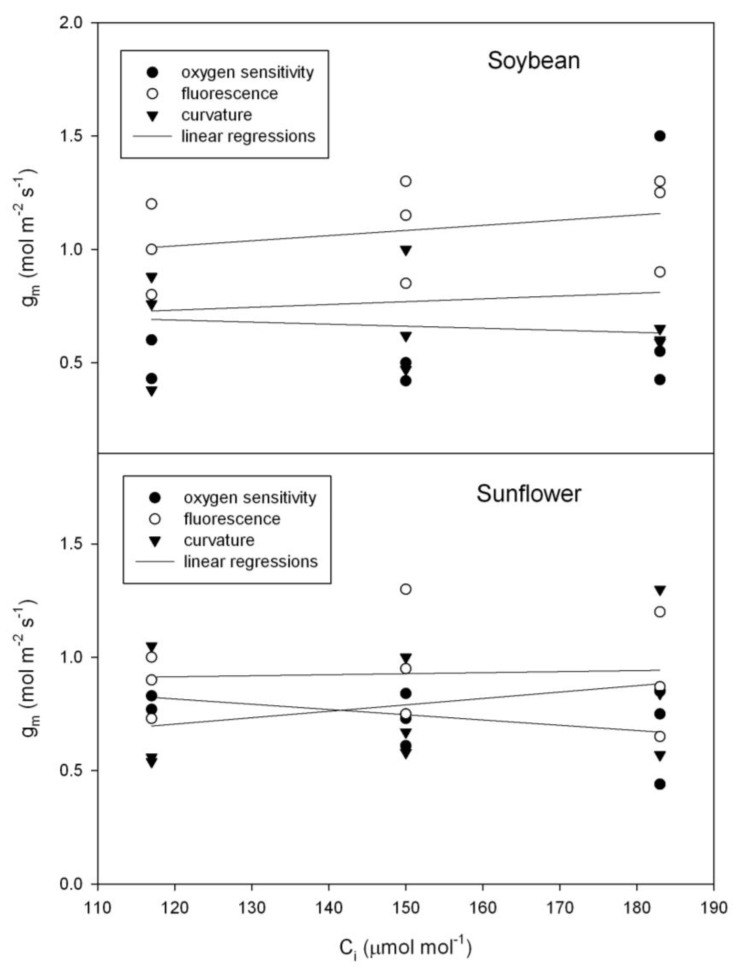
Mesophyll conductance to CO_2_ (g_m_) as a function of sub-stomatal CO_2_ concentration (C_i_) in soybean and sunflower. Mesophyll conductance was measured using three methods: the oxygen sensitivity of photosynthesis, variable J fluorescence, and the curvature of the Rubisco-limited A vs. C_i_ curve. See text for details.

**Figure 4:**
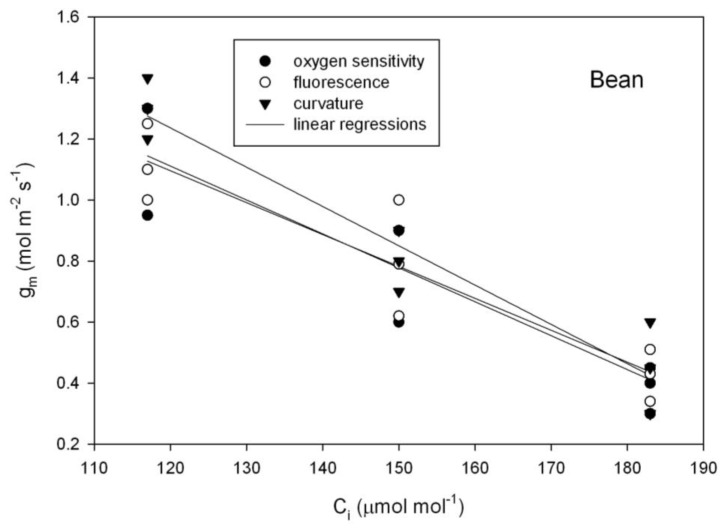
Mesophyll conductance to CO_2_ (g_m_) as a function of sub-stomatal CO_2_ concentration (C_i_) in common bean. Mesophyll conductance was measured using three methods: the oxygen sensitivity of photosynthesis, variable J fluorescence, and the curvature of the Rubisco-limited A vs. C_i_ curve. See text for details.

Although there was considerable leaf to leaf variation in g_m_, for both sunflower (*Helianthus annuus*) and soybean (*Glycine max*), there was no evidence of a change in g_m_ with C_i_ using any of the three independent methods of estimating g_m_ (Figure 3), as tested using repeated measures analysis of variance. In contrast with those species, in bean (*Phaseolus vulgaris*), all three methods of estimating g_m_ indicated a significant decrease in g_m_ with C_i_ over the 117 to 183 μmol mol^−1^ range of C_i_ (Figure 4).

## 3. Discussion

All three of the methods of estimating g_m_ used here are based on a biochemical model of C_3_ photosynthesis. However, the sensitivity of each of the three methods to model parameter values differs substantially. For example, the O_2_ sensitivity method was more sensitive to errors in the CO_2_ compensation point than to respiration or to Michaelis constants for CO_2_ or O_2_ [8], while the variable J fluorescence method was quite sensitive to errors in J, especially when C_i_ was less than about 80 mol mol^−1^ [6]. Sensitivity analyses are presented in the original references to the methods. Singh and Reddy [10] compared several variations of fluorescence-based methods with the O_2_ sensitivity method, and with the estimate based on the curvature of the initial slope of A vs. C_i_, and found only minor differences in g_m_ estimates among methods. However, that comparison used soybeans, where g_m_ does not change with C_i_. 

Many prior measurements of responses of g_m_ to C_i_ using variable J fluorescence were only considered to produce reliable results at C_i_ values of about 200 μmol mol^−1^ and higher, (e.g., [12,13,14]) based on the criterion of Harley et al. [6]. Any changes in g_m_ with C_i_ in the region of A vs. C_i_ curves in which Rubisco no longer limits A are less important to A, since A becomes much less sensitive to CO_2_ availability at high C_i_ than it is in the Rubisco-limited range [21]. The method of estimating g_m_ based on O_2_ sensitivity of A is most sensitive in the Rubisco-limited range of A vs. C_i_ curves [8]. The method based on the curvature of A vs. C_i_ curves was developed for the Rubisco-limited region [7]. The observations presented here also provided a test of the reliability of the variable J fluorescence method of estimating g_m_ at low C_i_ in these species. Unstressed leaves of C_3_ species often operate at C_i_ values at the upper end of the Rubisco-limited region of A vs. C_i_ curves [21], with generally lower C_i_ values in stressed leaves. This makes estimates of g_m_ in the Rubisco-limited region especially relevant.

All three methods of estimating g_m_ used here involve measurements of gas exchange at 21% O_2_, hence are potentially affected by photorespiration and CO_2_ transfer among cellular compartments, and are therefore potentially subject to the errors described by Tholen et al. [17]. The constant g_m_ reported here for soybean and sunflower indicate that those potential errors in g_m_ do not always occur even at low measurements C_i_, while previous measurements of g_m_ at low O_2_ indicate that photorespiration is not necessary to observe decreases in g_m_ with increasing C_i_ [12,18]. Thus, it remains unclear how important the potential artefactual decreases in g_m_ with increasing C_i_ identified by Tholen et al. [17] may be in general, although they did affect the estimates of g_m_ in soybean and sunflower in this experiment.

The lack of substantial change in g_m_ with C_i_ in soybean and a decrease in g_m_ with increasing C_i_ in bean has been previously reported for different cultivars of those species, using only the oxygen sensitivity method [15]. The two additional methods used here qualitatively agreed with those results. While significant intraspecific variation in g_m_ values have been found in soybean, and other species c.f. [22], the variable prior results for sunflower are difficult to reconcile. Vrabl et al. [14] reported strongly decreasing g_m_ with C_i_ in sunflower both for control leaves and for leaves treated with abscisic acid, while Schaufele et al. [23] found no variation in g_m_ with C_i_ for unstressed plants, but also found that application of abscisic acid resulted in large decreases in g_m_ with increasing C_i_. Qiu et al. [24] also found a large effect of abscisic acid on g_m_ in raspberry. Our results for this sunflower cultivar were similar to those of Schaufele et al. [23], with no change in g_m_ with C_i_ for unstressed plants over the limited, low C_i_ range tested here. Several papers Page: 6 (e.g., [12,25,26,27]) have suggested that an influence of aquaporins on CO_2_ transport and g_m_ might provide a mechanism for changes in g_m_ with C_i_, such as observed here in bean. It seems possible that differences among species as to whether g_m_ changes with C_i_ might be related to the predominance of physical diffusion processes in g_m_ in some species and a larger contribution of metabolic factors in other species. The bean vs. soybean contrast in g_m_ sensitivity to C_i_ could be a useful experimental system to understand species differences in sensitivity.

The new method described here of ramping CO_2_ to rapidly obtain A vs. C_i_ curves could also be useful in many other situations, such as comparisons among genotypes or treatments in photosynthetic parameters. In our experiments, and perhaps in other applications of CO_2_ ramping, it was prudent to let CO_2_ increase until no further increase in photosynthesis was observed, even though this took a little extra time. During the ramping, it is very difficult to estimate immediately what the C_i_ value is at any point in time, so allowing CO_2_ to saturate photosynthesis ensures that sufficiently high C_i_ has been achieved. The rate of CO_2_ increase during ramping can be selected by the user. The rate used here was chosen so that stomatal conductance did not change significantly during the ramping for these species, simplifying post-processing of the data.

## 4. Materials and Methods

Soybean (*Glycine max* L. Merr., cultivar Holt), sunflower (*Helianthus annuus* L., cultivar Mammoth Gray Stripe) and common bean (*Phaseolus vulgaris* L., cultivar Red Hawk) were grown together in an indoor controlled environment chamber. The chamber air temperature was 25 °C, the dew point temperature was 18 °C, the photosynthetic photon flux density was 1000 μmol m^−2^ s^−1^ for 12 h per day from a mixture of metal halide and high-pressure sodium lamps, and CO_2_ concentration was controlled to 420 ± 20 μmol mol^−1^ for 24 h per day. Plants were grown one per pot in 15 cm diameter pots filled with vermiculite and flushed daily with a complete nutrient solution. Each measurement of the response of mesophyll conductance response to C_i_ was made on a different leaf from a different plant.

Most leaf gas exchange measurements were made with a CIRAS-3 photosynthesis system (PP Systems, Amesbury, MA, USA). The leaf cuvette had a 2.5 cm^2^ window, and light was provided by red, green and blue light-emitting diodes, set for 38% red, 37% green, and 25% blue, as the closest approximation to sunlight. For estimation of g_m_ based on fluorescence, the instrument was equipped for simultaneously measuring chlorophyll fluorescence. Measurements of g_m_ based on the oxygen sensitivity of photosynthesis [8] were made both with the CIRAS-3 and with a LiCor 6400 XT photosynthesis system (LiCor, Inc., Lincoln, NE, USA) in order to test the sensitivity of CO_2_ and H_2_O analysis to O_2_ in the CIRAS-3. The LiCor system had a cuvette window of 6 cm^2^ area, and used the LiCor red and blue light emitting dioxide light unit. The O_2_ sensitivity of CO_2_ and H_2_O analysis in the LiCor 6400 XT is known, and corrections to outputs based on O_2_ are built into the instrument operating system. The sensitivity to O_2_ of the CIRAS-3 system was not known.

Three independent methods of estimating g_m_ were used. The curvature of the A vs. C_i_ curve in the Rubisco-limited region was used to estimate g_m_, using the gas exchange calculation utility of Sharkey et al. [20]. In order for this method to be applied for different Rubisco-limited C_i_ ranges, a new system was developed for rapidly collecting very dense data points. The CIRAS-3 system utility “stored differential-balance” was used to store the change in sensitivity of CO_2_ and H_2_O to background CO_2_ and H_2_O for the anticipated ranges of each variable. These values are quite stable over time (days), in the CIRAS-3. The CIRAS-3 system utility program which controls linear increases in reference CO_2_ was set to increase the reference CO_2_ from 100 μmol mol^−1^ at a rate of 233 μmol mol^−1^ min^−1^ following an initial 2 min period of constant concentration of 100 μmol mol^−1^. Data were stored approximately every 2 s during the increase in reference CO_2_. First, this CO_2_ ramping program was run with an empty cuvette. Then it was run with leaves in the cuvette. Leaf temperature was controlled at 25 ℃, the water vapor pressure deficit (VPD) was between 1.0 and 1.5 kPa, and the photosynthetic photon flux density (PPFD) was 1500 μmol m^-2^ s^−1^. Measurements on leaves were terminated when the “apparent” A values no longer increased with reference CO_2_. For each time step in the data files, the “A” value for the empty cuvette was subtracted from the value when the leaf was present. This is similar to the approach of Stinziano et al. [28], although their method of obtaining corrected values of A was more complex, as necessary with the instrument used. From the corrected A values, C_i_ was re-calculated in the usual way from stomatal and boundary layer conductances, A, and external CO_2_ [21], using the corrected values of A. Stomatal conductance never changed substantially during the CO_2_ ramping procedures. As a test of this new method of developing A vs. C_i_ curves using rapid CO_2_ ramping, A vs. C_i_ curves obtained with ramped CO_2_ were compared with curves on the same leaves obtained with the same instrument using traditional steady-state gas exchange measurements at several steps of external CO_2_ under the same conditions of PPFD, leaf temperature, and VPD as used in the CO_2_ ramping. For tests of changes in g_m_ with C_i_, A vs. C_i_ curves obtained by CO_2_ ramping were arbitrarily separated into four successive sections, from C_i_s of 100 to 133, 134 to 166, and from C_i_s of 167 to 200, and all C_i_s above 200 μmol mol^−1^. The three lower parts of the low C_i_ curves were separately combined with the fourth, upper C_i_ part. The Sharkey et al. utility [20] was then used to estimate g_m_ separately for the three lower C_i_ sections with uniform upper C_i_ data, assuming limitation by the maximum carboxylation capacity of Rubisco (V_Cmax_) for the three lower C_i_ ranges, and triose phosphate utilization (TPU) limitation at the highest Ci. The division between the V_Cmax_ and electron transport (J)-limited regions was done by minimizing the error term. The utility program values for temperature dependencies of parameters were used, and the utility was used to estimate respiration. Three leaves from different plants of each species were used to develop these tests of variation of g_m_ with C_i_.

The second method used was the “variable J” method combining leaf gas exchange and fluorescence [6]. Steady state A vs. C_i_ curves were obtained, using sequential external CO_2_ concentrations of 400, 100, 150, 200, 250, 300, 400, 500, 600, 800, 1000, and 1800 μmol mol^−1^. Leaves were measured at the same PPFD, leaf temperature (T) and VPD as previously described. Light was supplied by red, green and blue light-emitting diodes, set at 38% red, 37% green, and 25% blue as a close approximation to sunlight. Leaf absorption was assumed to be 0.84, and the fraction absorbed by photosystem II was assumed to be 0.5. At each CO_2_ level, Phi PSII and J were obtained using Multi-Pulse^TM^ fluorescence measurements [29]. The measurement at the highest CO_2_ (“non-photorespiratory” conditions) was used to determine the proportionality between J and fluorescence yield [6]. Because photorespiration at the highest CO_2_ concentration could theoretically have still been 2 to 3% of photosynthesis [30], additional estimates of g_m_ were made using adjusted values of the proportionality between J and fluorescence yield assuming photorespiration was 2.5% of photosynthesis. These measurements were made on three leaves from different plants of each species. Values of g_m_ for each leaf were obtained in the same three C_i_ regions as used in the previous method.

The third method used, the oxygen sensitivity of photosynthesis method of estimating g_m_ [8] was implemented by developing A vs. C_i_ curves of the same leaf both at 21 and 2% O_2._ Leaf temperature, VPD, and PPFD were the same as in the prior methods. The A vs. C_i_ curves at 2% and 21% O_2_ were used to calculate g_m_ at C_i_ values ranging from below 100 to about 200 μmol mol^−1^, by solving for g_m_ values compatible with A vs. C_i_ values at both O_2_ concentrations [10]. Values of respiration in the light were determined by extrapolating A vs. C_i_ curves at 2% O2 to 0 C_i_. The calculation utility of Singh and Reddy [10] uses the same other biochemical parameter values as in the Sharkey et al. utility [20]. Values of A vs. C_i_ at 2% O_2_ estimated using the LiCor 6400 XT instrument with correction for O_2_ were compared with A vs. C_i_ values estimated using the CIRAS-3 instrument without correction for O_2_ concentration. The comparisons between instruments were made using opposite sides of the same leaves for two individual plants each of sunflower and soybean. The response of g_m_ to C_i_ using the O_2_ sensitivity method with the CIRAS-3 system was then determined for three leaves from different plants of each species, and summarized for the same three ranges of C_i_ as used in the curvature method.

Repeated measures analysis of variance was used separately for each method for each species to test whether g_m_ changed with the C_i_ range.

## 5. Conclusions

The results presented here indicate that the variable J fluorescence method of estimating g_m_ may be valid at lower C_i_ values than often assumed because it agreed with two other methods, which are especially suited to measurements of g_m_ in the Rubisco-limited region of A vs. C_i_ curves. Whether g_m_ varies with C_i_ in the Rubisco-limited range depends upon the species, but three different methods of estimating g_m_ were in agreement regarding whether g_m_ changed with C_i_ or was constant.

* Mention of specific brands of instruments does not constitute endorsement of those brands of instruments by the USDA to the exclusion of others which may be suitable.
